# Effects of a High-Glucose Diet on *Caenorhabditis elegans* Key Biological Functions and Survival to Bacterial Infections

**DOI:** 10.1021/acsomega.5c12287

**Published:** 2026-05-09

**Authors:** Belisa Avila Rodrigues, Amanda Moreira de Barros, Júlia Machado Menezes, Luisa Jacqueline Maboni, Eduardo Rigon Zimmer, Karine Rigon Zimmer

**Affiliations:** † Laboratory of Biofilms and Alternative Models (BioModal), Universidade Federal de Ciências da Saúde de Porto Alegre (UFCSPA), 245 Sarmento Leite Street, Porto Alegre, Rio Grande do Sul 90050-170, Brazil; ‡ Postgraduate Program in Biosciences, Universidade Federal de Ciências da Saúde de Porto Alegre (UFCSPA), 245 Sarmento Leite Street, Porto Alegre, Rio Grande do Sul 90050-170, Brazil; § Postgraduate Program in Biological Sciences: Biochemistry and Postgraduate Program in Pharmacology and Therapeutics, Universidade Federal do Rio Grande do Sul (UFRGS), 2600 Ramiro Barcelos Street, Porto Alegre, Rio Grande do Sul 90035-002, Brazil

## Abstract

The widespread consumption of high-glucose diets and the rise of antimicrobial resistance underscore the importance of understanding how such diets influence host organisms and their immune responses to bacterial pathogens. In this study, we examine the effects of a high-glucose diet on key biological functions of the nematode *Caenorhabditis elegans*, as well as its response to infections with*Staphylococcus aureus* and*Pseudomonas aeruginosa*after being fed the same diet. To date, it is the first study to provide evidence of an association between a high-glucose diet and*P. aeruginosa*infection. Worms were fed a 2% glucose diet from the L1 to L4 developmental stages. Results showed that worms on the high-glucose diet exhibited increased defecation rate, elongated body length, reduced progeny production, greater lipid droplet accumulation, elevated *gst-4* expression, and changes in ROS and senescence pigment production compared to controls. Additionally, the proportion of worms with dopaminergic neuron damage was higher in the high-glucose group. DAF-16 cellular localization remained unaffected. Interestingly, worms previously exposed to a high-glucose diet demonstrated increased median survival upon infection with*S. aureus* and*P. aeruginosa*relative to controls. Furthermore, the high-glucose diet conferred protection of the intestinal barrier against*P. aeruginosa* infection and enhanced the survival of the immune-deficient*C. elegans* RB1573 strain infected with*P. aeruginosa*. These findings highlight the impact of a high-glucose diet on essential biological functions in*C. elegans* and reveal a protective effect against bacterial infections.

## Graphical abstract

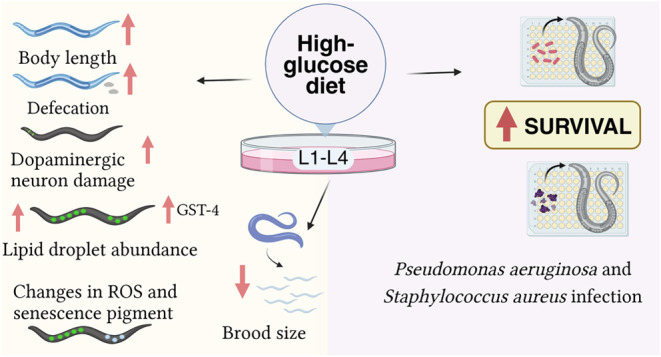

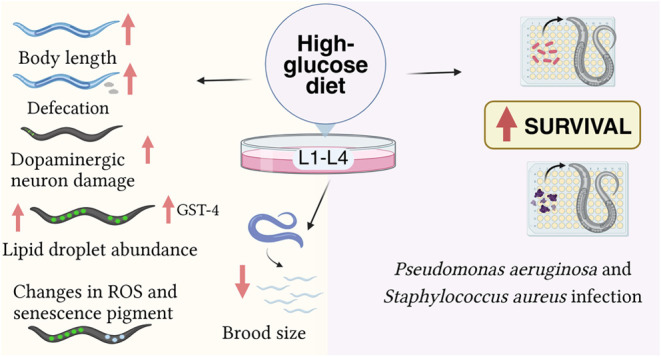

## 1 Introduction

In recent decades, there has been a high consumption of carbohydrate-rich diets. These diets can increase glucose levels in the body, leading to metabolic disorders that may impact longevity and immunity in various organisms, including insects, worms, and mammals.^1–3^ Recently, a study using*Drosophila melanogaster* found that a high-glucose diet increased susceptibility to infections by*Providencia rettgeri* and*Serratia marcescens*.^4^

Nevertheless, many aspects of the association between high-glucose diets and the response to bacterial infections require further investigation. Given the challenges posed by antimicrobial resistance and the widespread consumption of glucose-rich diets, it is crucial to investigate the effects of high-glucose diets on the body and how these diets may impact responses to bacterial infections. A deeper understanding of the relationship between diet and bacterial infections could lead to new prevention and treatment strategies.^5,6^

*Caenorhabditis elegans* is a very suitable model for these investigations due to its low maintenance cost, small size, short life cycle, production of a large number of offspring, and complete genome sequencing.^7^ Furthermore, worms have a highly characterized nervous system, and due to its transparency and the construction of GFP-labeled strains, it is possible to visualize neuronal circuits in the worms and assess potential damage to them.^8^

The worm’s response to bacterial infections involves numerous conserved cellular signaling pathways, such as p38 MAPK, insulin/IGF-1 signaling, and DBL-1/TGF-β, the production of antimicrobial peptides, and the release of reactive oxygen species (ROS).^9^ DAF-16 and SKN-1 transcription factors are important effectors in the defense against bacterial pathogens. Both are activated under stress conditions and during host–pathogen interaction, when they are translocated from the cell’s cytoplasm to the nucleus, inducing the expression of innate immune genes.^10,11^

Moreover, the nervous system influences worms’ responses to bacterial infection by regulating immunity. Neurotransmitters play a role in controlling intestinal responses to bacterial pathogens. The dopaminergic system, for example, can negatively control immune defenses in*C. elegans*.^12,13^

Several studies have been using *C. elegans* to evaluate bacterial pathogenesis, since many human pathogens also infect worms, often using the same virulence factors.^14^ *Pseudomonas aeruginosa* and*Staphylococcus aureus*are known for difficult-to-treat infections, especially in hospital settings, and are considered high-priority pathogens in the latest WHO Bacterial Priority Pathogens List.^15^

*S. aureus*infection in*C. elegans* begins with intestinal colonization, and as the infection progresses, it causes intestinal cell lysis and whole-body cellular damage, leading to death. In*P. aeruginosa* infections, in addition to intestinal colonization, the production of molecules such as pyoverdine and toxins also impairs worms’ body functions, causing cellular damage and leading to death.^16,17^

In worms, high-sugar diets have been associated mainly with decreased longevity and with changes in reproduction, learning, and neurodegeneration.^3,18–24^ Two studies directly linked bacterial infection and glucose diets, but they yielded opposing outcomes in*C. elegans*. In one study, a high-glucose diet (0.5%) had a negative influence on the response to *Salmonella typhimurium* infections. In another study, a high-glucose diet (2%) had a positive effect on the response to*S. aureus* and*Escherichia coli* infections.^25,26^ To date, no study has evaluated the association between*P. aeruginosa* infection and glucose diet in*C. elegans*.

Thus, this work aimed to investigate the effects of a 2% high-glucose diet on key biological functions of*C. elegans* and the response to bacterial infections with*S. aureus* and*P. aeruginosa*. The high-glucose diet impacted key biological functions in worms, including defecation, body size, brood size, and dopaminergic neuron integrity. High-glucose-fed worms also exhibited higher lipid droplet abundance, increased *gst-4* expression, changes in ROS production and senescence pigment formation. The diet also protected the worms from*S. aureus*and*P. aeruginosa* infections.

## 2 Results

### 2.1 Effects of the High-Glucose Diet on*C. elegans* Key Biological Functions

We measured the pumping per minute (ppm) of worms fed with a high-glucose diet and compared it to control worms without glucose. Worms fed with the high-glucose diet did not show a statistically significant difference in pharyngeal pumping (274.5 ppm) compared to worms fed with the control diet (267.3 ppm), demonstrating that the high-glucose diet did not impact worm feeding (Figure 1A).

**Figure 1:**
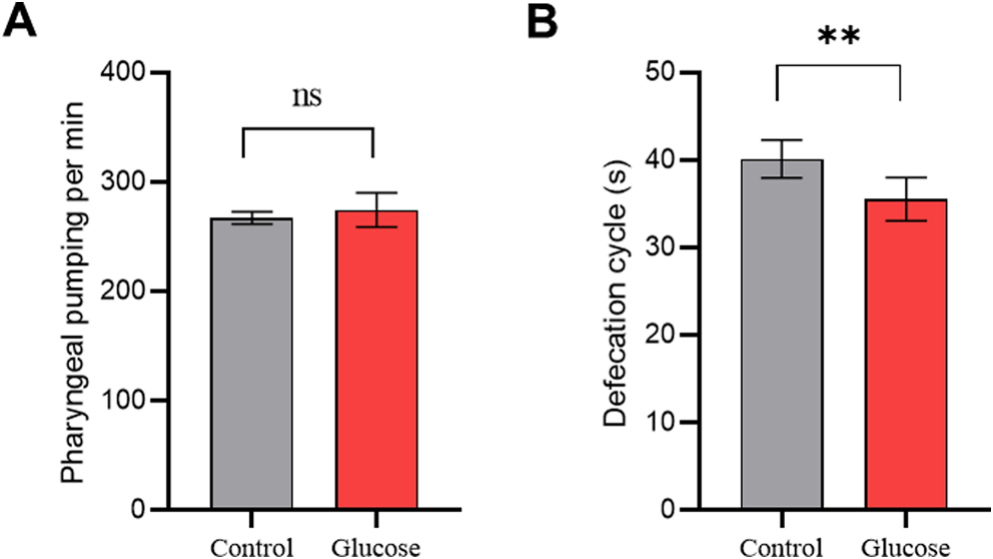
A high-glucose diet did not affect pharyngeal pumping but decreased the time of the defecation cycle. (A) Mean number of pharyngeal pumping per minute of worms fed a control diet (Control), and worms fed a high-glucose diet (Glucose). (B) Mean time in seconds between three worms’ defecation when fed the control diet (Control) and high-glucose diet (Glucose). For (A, B), assays were performed seven times independently and in duplicate (*N* = 70). Differences between groups (glucose and control) were analyzed using Student’s *t* test, ns: not significant, **p: 0.0032.

However, the high-glucose diet affected the defecation cycle of worms. Worms fed glucose had a decrease in the time of the defecation cycle compared to control worms. The mean time between defecation contractions was 35 s in glucose-fed worms and 40 s in control worms (Figure 1B).

Since nutrients are necessary for the growth of worms, high-glucose diets could influence worm body size. In our work, the glucose intake affected the body length of worms, in which worms fed glucose were approximately 12% longer than the control worms (Figure 2A). However, no statistical differences were found in the body area between worms fed glucose or control food (Figure 2B).

**Figure 2:**
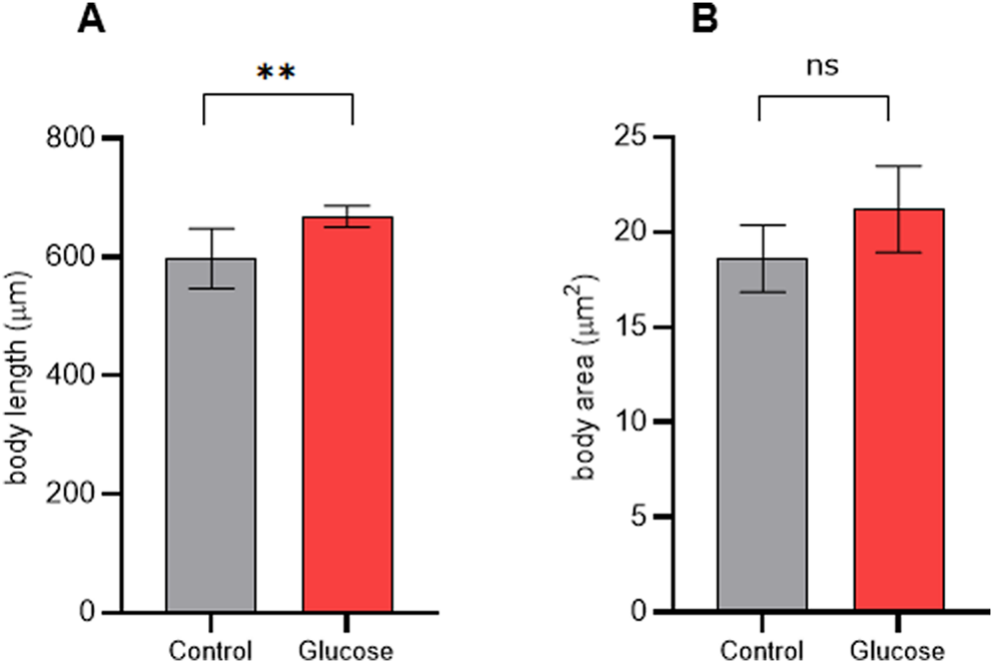
Worms fed a high-glucose diet are longer than worms fed a control diet. (A) Body length mean in μm of worms fed a high-glucose diet (Glucose) and control-fed worms (Control). (B) Body area mean in μm^2^ of worms fed a high-glucose diet (Glucose) and control-diet-fed worms (Control). In (A, B), assays were performed at least three times independently and in duplicate. Differences between groups (glucose and control) were analyzed using Student’s *t* test, **p: 0.0088, ns: not significant.

Furthermore,*C. elegans* brood size was negatively affected by a high-glucose diet. We measured the number of progeny from worms fed glucose until the L4 stage and compared it with that of control worms. The previous high-glucose diet led to a reduction of 27% in the number of progenies compared to the control (Figure 3).

**Figure 3:**
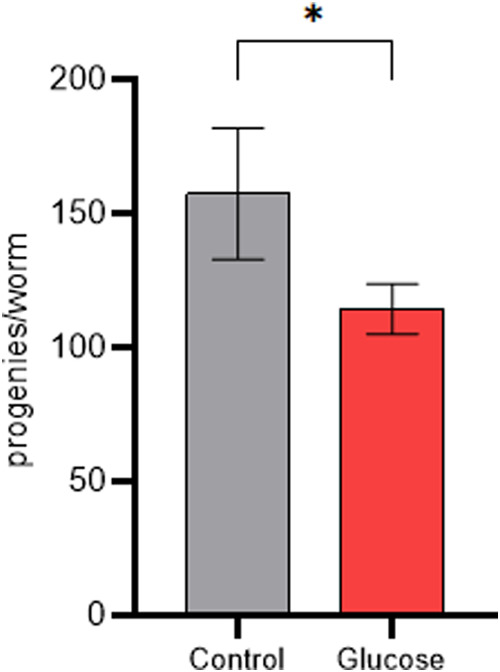
High-glucose diet decreased the brood size of worms. Mean number of progenies per worm fed a high-glucose diet (Glucose) and fed a control diet (Control). Brood size assay was performed three times independently. Differences between groups (glucose and control) were analyzed using Student’s *t* test, *p: 0.0165.

Using the GFP-tagged strain, BY200 (Figure 4A), we performed an analysis of dopaminergic neurons of worms fed a high-glucose diet and compared it with worms fed a control diet. We counted the number of worms that presented at least one type of neuron damage and found that the number of worms that presented neuronal damage was higher when worms were fed a high-glucose diet (58.9%) than when fed a control diet (36.7%) (Figure 4B).

**Figure 4:**
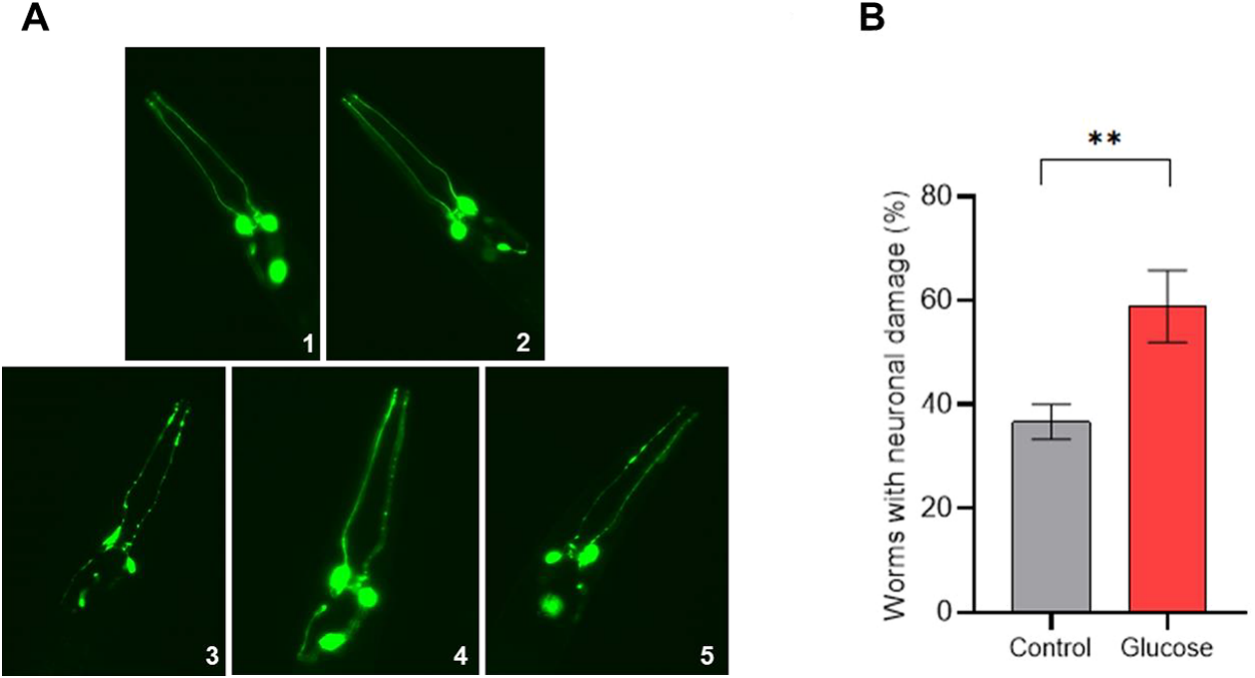
Dopaminergic neuron damage analysis. (A) Representative images of worms without dopaminergic neuron damage (1 and 2) and worms with dopaminergic neuron damage (3–5) that were found in both groups. Edited with ImageJ software for better visualization. (B) The percent of worms with any dopaminergic neuron damage is higher with a high-glucose diet than with a control diet. Mean percent of worms that had at least one type of dopaminergic neuron damage after a high-glucose diet (Glucose) and a control diet (Control). Thirty images from each group (glucose and control) were analyzed from three independent assays. Differences between groups (glucose and control) were analyzed using Student’s *t* test, **p: 0.0075.

### 2.2 Lipid Droplet Abundance and *gst-4* Expression

In our work, we utilized Nile Red dye to measure lipid droplet abundance in the worm body (Figure 5A). Fluorescence intensity of individual worm bodies was measured (Figure 5B), and then we performed a CTCF calculation to take into account the body area of the animals. As shown in Figure 5C, worms that were fed a high-glucose diet showed an increase in lipid droplet abundance compared to normally fed worms.

**Figure 5:**
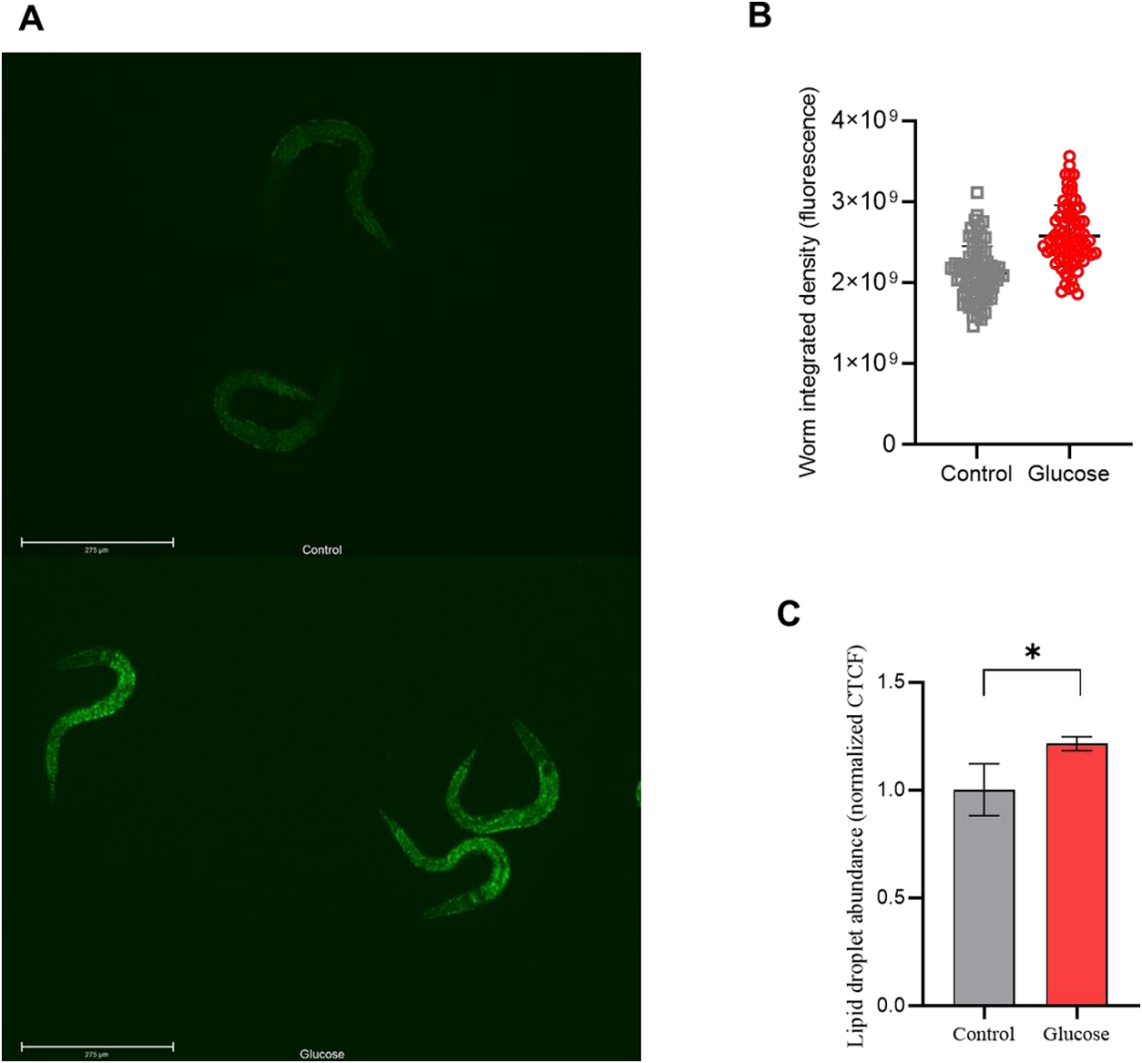
Worms fed a high-glucose diet had more lipid droplet abundance than control-diet-fed worms. (A) Representative images of *C. elegans* N2 dyed with Nile Red. Edited with ImageJ software for better visualization. Scale bar: 275 μm. (B) Representation of individual worm bodies’ fluorescence that was used to calculate the CTCF (Worm integrated density). (C) Lipid droplet abundance of worms fed a high-glucose diet (Glucose), and worms fed a control diet (Control). Results were expressed as area-corrected worm fluorescent intensity (CTCF) normalized by control. Four independent assays with 20 worms were performed (*N* = 80). Differences between groups (glucose and control) were analyzed using Student’s *t* test, *p: 0.0138.

GST-4 (Glutathione S transferase 4) is an enzyme involved in Phase II detoxification in *C. elegans*.^27^ We quantify GST-4 gene (*gst-4*) expression through fluorescence intensity (Figure 6A,B), using the same approach as lipid droplet abundance (CTCF calculation). The results showed a slight increase in *gst-4* expression in worms fed with a high-glucose diet compared to worms fed the control diet (Figure 6C).

**Figure 6:**
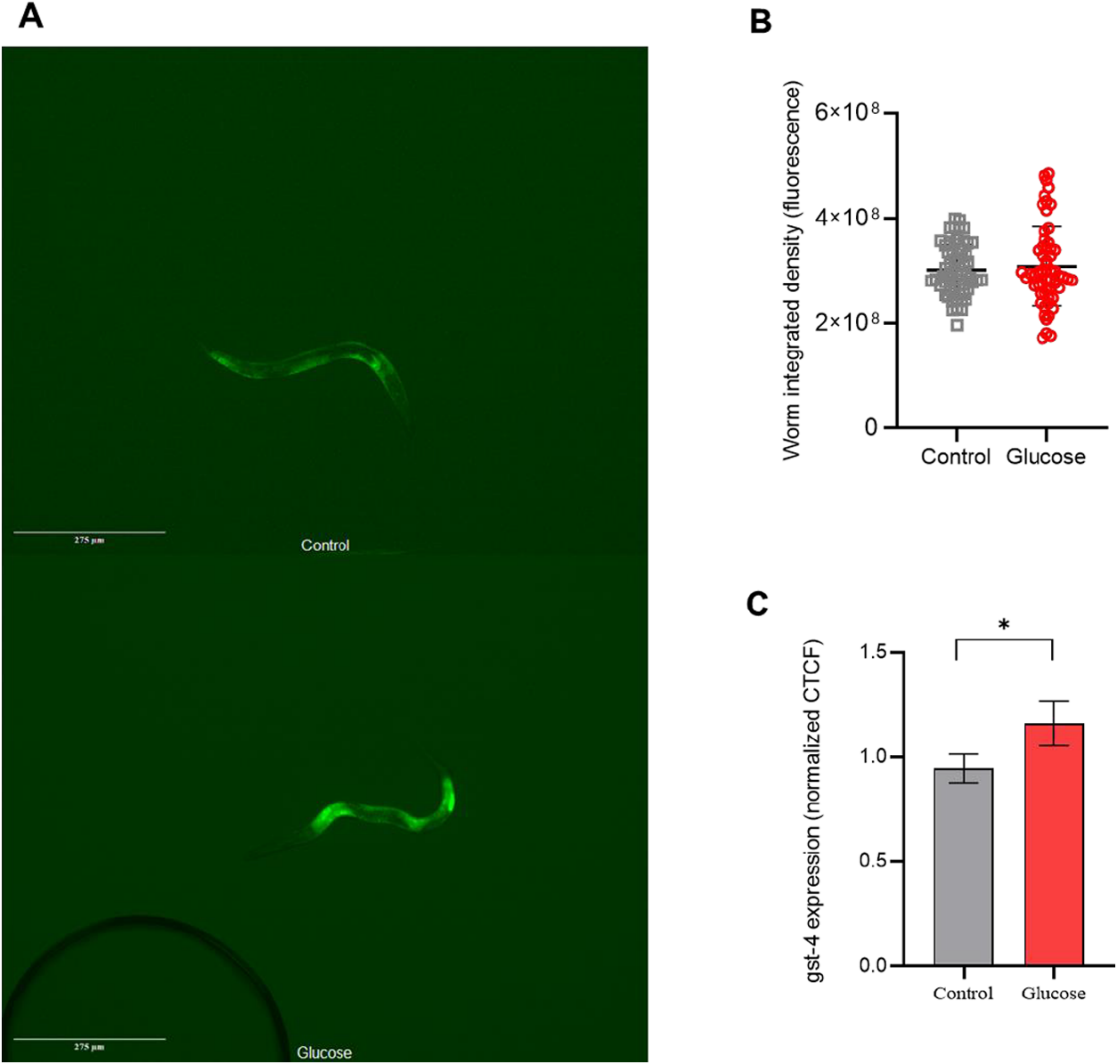
Worms fed a high-glucose diet had more *gst-4* fluorescence expression than control-diet-fed worms. (A) Representative images of *C. elegans* CL2166. Edited with ImageJ software for better visualization. Scale bar: 275 μm. (B) Representation of individual worm bodies’ fluorescence that was used to calculate the CTCF (Worm integrated density). (C) *gst-4* fluorescence expression of worms fed a high-glucose diet (Glucose), and worms fed a control diet (Control). Results were expressed as area-corrected worm fluorescent intensity (CTCF) normalized by control. Three independent assays with 20 worms were performed (*N* = 60). Differences between groups (glucose and control) were analyzed using Student’s *t* test, *p: 0.0421.

### 2.3 ROS Production and Senescence

The production of reactive oxygen species (ROS) is an important marker of oxidative stress and is also associated with the response against pathogens in worms.^28^ We used H_2_DCFDA to measure the ROS production of L4 worms after the high-glucose diet and the control diet along time. The initial times analyzed (0 h and 6 h) showed no statistical differences between the groups. However, in late time points (24 h and 48 h), ROS production was higher in worms fed the control diet than worms previously fed the high-glucose diet (Figure 7A).

**Figure 7:**
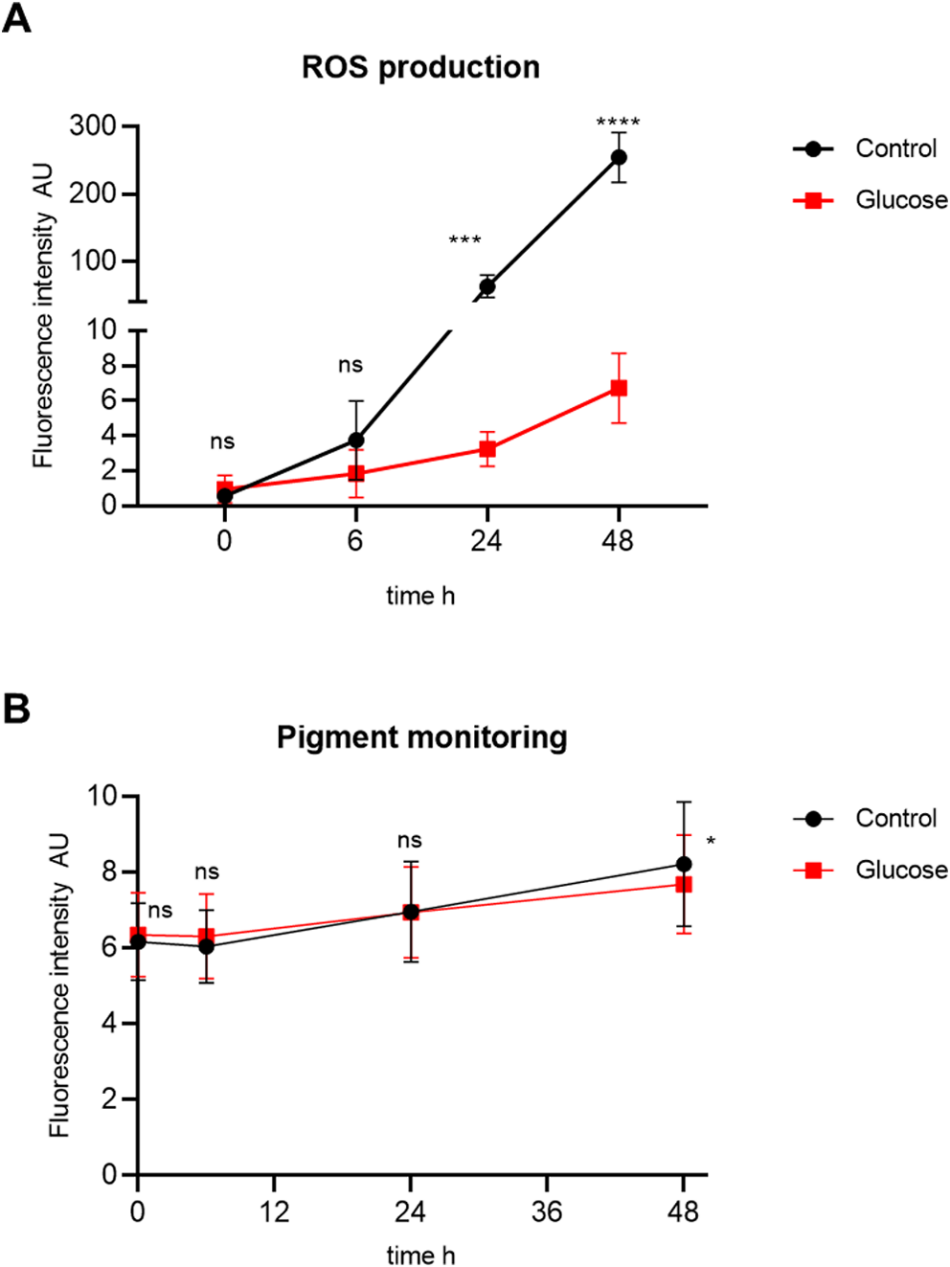
ROS production and senescence evaluation of worms previously fed a high-glucose diet and a control diet. (A) ROS production over time by worms previously fed a high-glucose diet and a control diet. (B) Autofluorescent blue pigment monitoring over time (AGE formation). For (A, B), data was represented by mean (±SD) of fluorescence intensity (AU). At least four technical replicates from 7 independent assays were performed (*N* = ∼2800). Difference between groups (glucose and control) were analyzed by two-way ANOVA test followed by Sidak’s multiple comparisons test. ns: not significant; *** *p*: 0.0003; ****p < 0.0001; *0.0108.

Senescence was evaluated by monitoring a blue autofluorescence pigment related to the formation of advanced glycation end products (AGEs), over time. Worms previously fed both diets showed similar AGE formation, with no statistical differences. At the last time point evaluated (48 h), however, a statistical difference was observed, with worms fed the control diet showing slightly higher AGE formation than worms fed the high-glucose diet (Figure 7B).

### 2.4 DAF-16 Cellular Localization

We investigate the impact of the high-glucose diet on the nuclear localization of the transcription factor DAF-16, using the*C. elegans*strain TJ356 (Figure 8A). The findings indicated that a higher percentage of worms were categorized as intermediate in both the high-glucose diet (68.3%) and the control diet (77%). DAF-16 nuclear localization was identified in 26.3% of worms fed a high-glucose diet and 3.6% in control worms. Cytoplasmic localization was identified in 5.3% of the worms fed a high-glucose diet and 19.3% of the control worms. However, when we compared worms fed a high-glucose diet with control worms for each cellular localization, no statistically significant differences were observed (Figure 8B).

**Figure 8:**
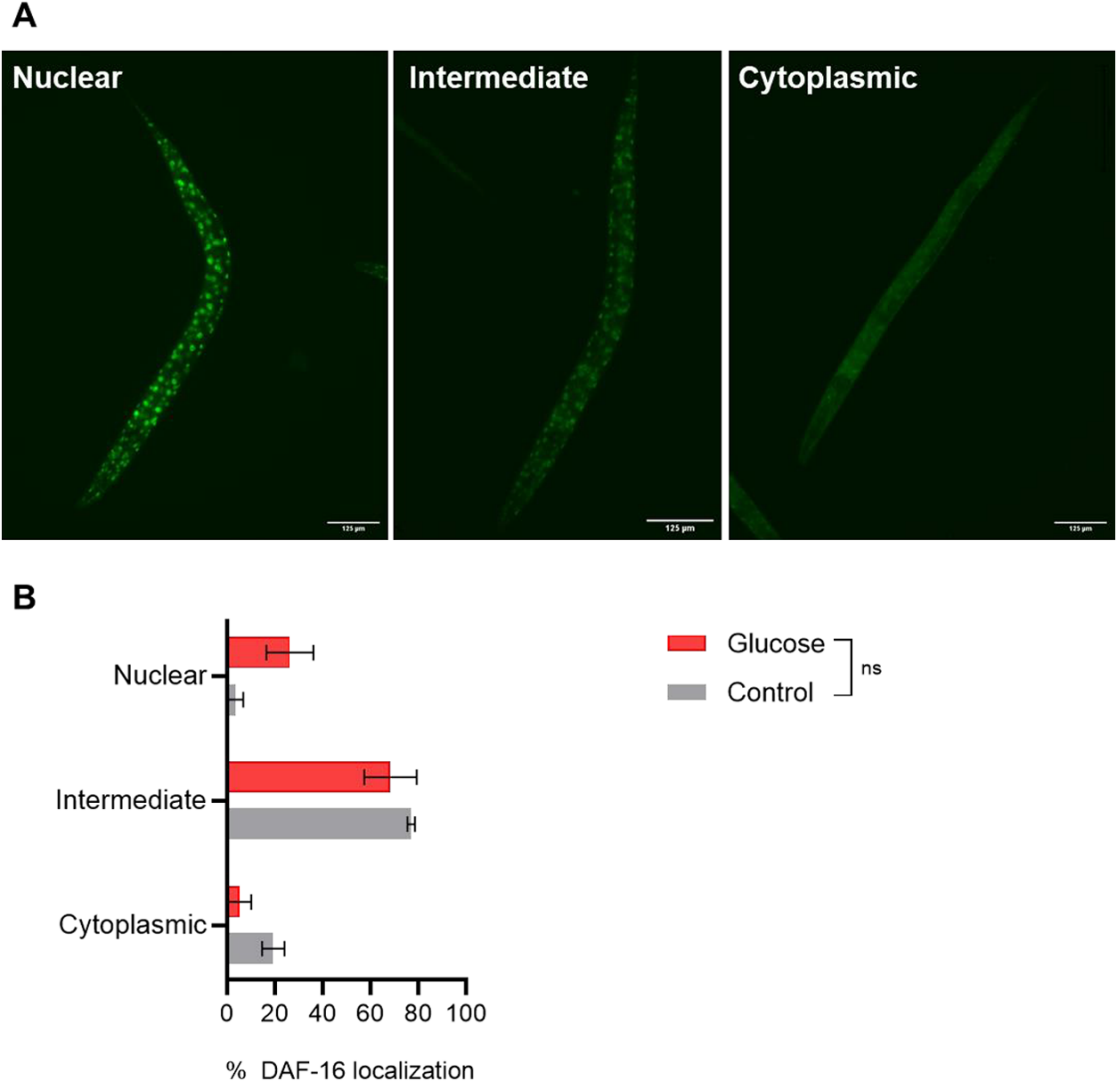
Cellular localization of DAF-16 in*C. elegans* TJ356 fed a high-glucose diet was predominantly intermediate. (A) Representative images of DAF-16 nuclear localization of the TJ356 strain, edited with ImageJ software for better visualization. Scale bar: 125 μm. (B) Mean percent of worms with DAF-16 nuclear, intermediate, and cytoplasmic localization after a high-glucose diet (Glucose) and a control diet (Control). Twenty images from each group (glucose and control) were analyzed from three independent assays (*N* = 60). Comparisons between glucose and control groups for each cellular location were performed using two-way ANOVA followed by Sidak’s multiple comparisons test, ns: not significant.

### 2.5 *S. aureus*and*P. aeruginosa*Infection

Bacterial infection of worms was performed using a liquid-killing assay, with the same medium conditions (M9 buffer supplemented with cholesterol and FUDR) as for*P. aeruginosa*and*S. aureus*infection. Images using GFP-tagged*E. coli* OP50 (Figure 9A,9B) and mCherry-tagged*P. aeruginosa* *PA14* (Figure 9C and 7D) were taken to confirm worm uptake of bacteria in these assay conditions. Using the Kaplan–Meier curve, we first compared worms exposed to the noninfected control (*E. coli* OP50) with the worms exposed to*S. aureus*and*P. aeruginosa* (Figure 10A–D) to ensure an infection process in both groups analyzed (glucose and control diets).

**Figure 9:**
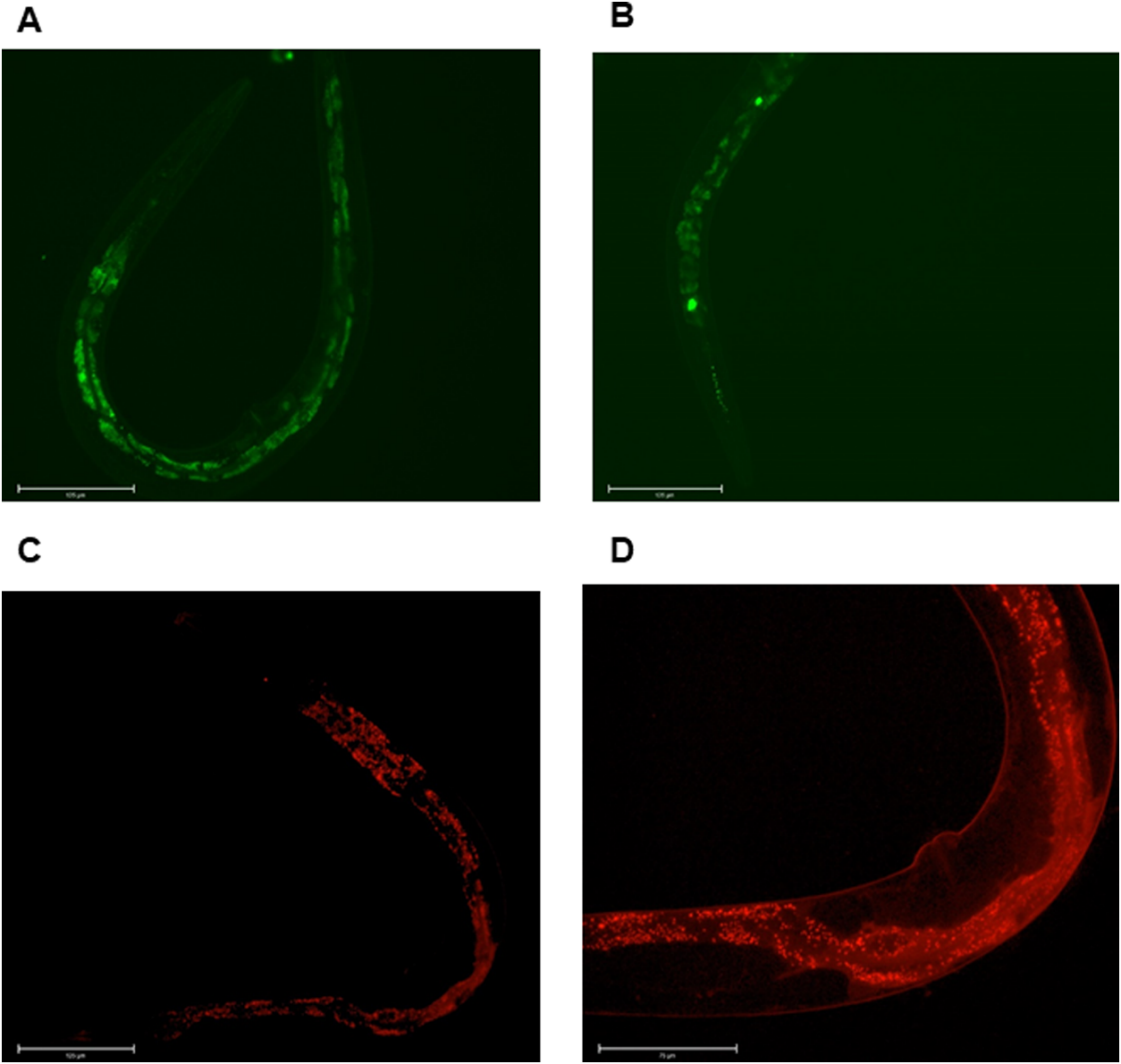
(A, B) Representative images of control-diet-fed*C. elegans*feeding with GFP-tagged*E. coli* OP50 from the liquid survival assay. (C) and (D) Representative images of control-diet-fed*C. elegans* infected with mCherry-tagged*P. aeruginosa*. (C) Whole-body worm and (D) midsection of the body, revealing a portion of the intestine. Photographed with EVOS FL Auto 2 Imaging System (Thermo Fisher Scientific) and edited with ImageJ software. Scale bar: 125 μm.

**Figure 10:**
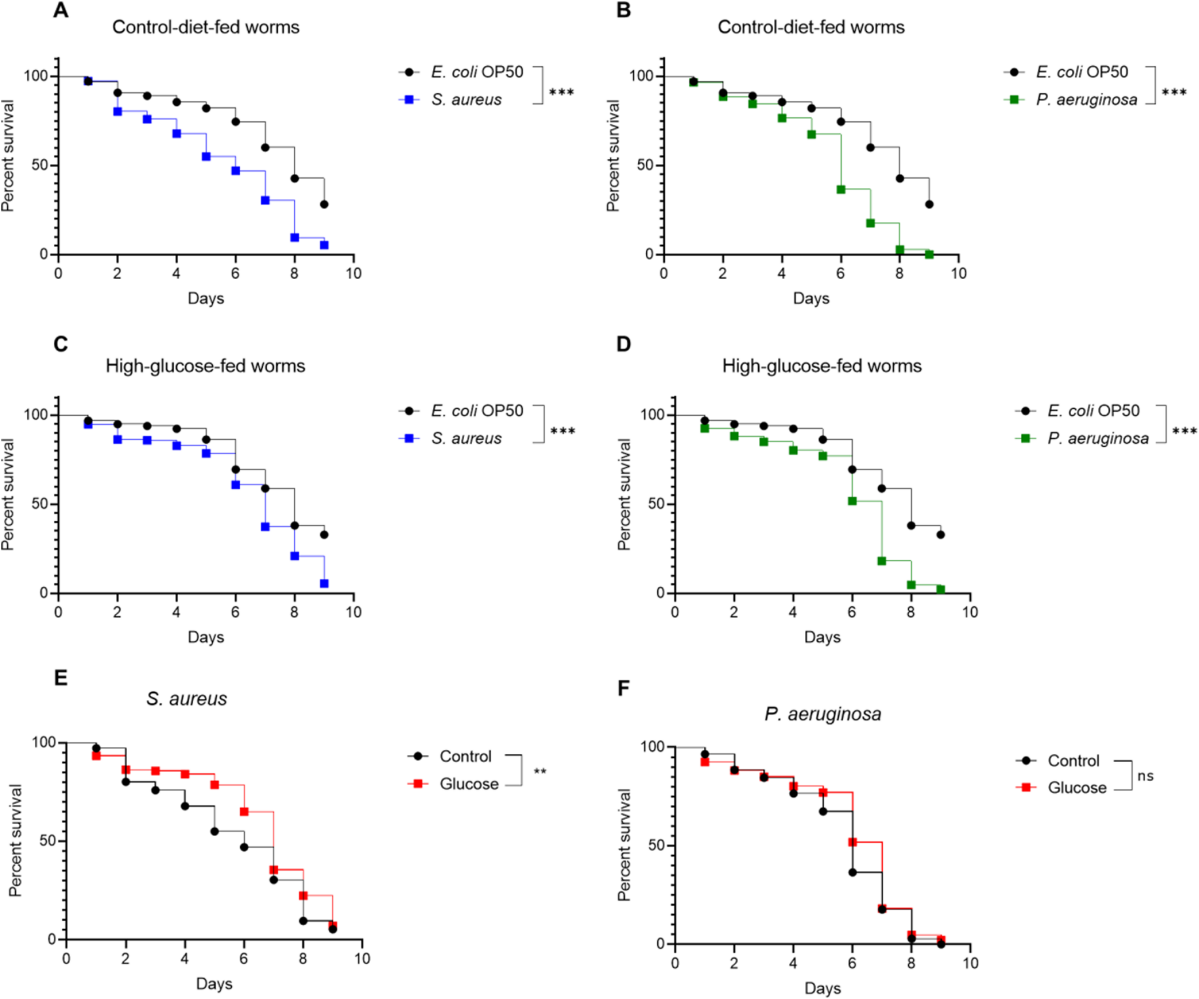
A 2% high-glucose diet increased worm survival in bacterial infection. Survival curves were compared using Log-rank (Mantel-Cox) and Gehan–Breslow–Wilcoxon tests. *P*-values were adjusted for multiple comparisons using the Holm-Bonferroni method. (A) *S. aureus*infection of control-diet-fed worms. Worms infected with*S. aureus*had a median survival of 6 days, and worms fed *E. coli*OP50 had a median survival of 8 days. Differences between curves were statistically significant (Log-rank test and Gehan–Breslow–Wilcoxon test; *p* < 0.0007). (B) *P. aeruginosa*infection of control diet worms. Worms infected with*P. aeruginosa*had a median survival of 6 days, and worms fed*E. coli*OP50 had a median survival of 8 days. Differences between curves were statistically significant (Log-rank test and Gehan–Breslow–Wilcoxon test; *p* < 0.0007). (C) *S. aureus* infection of high-glucose-fed worms. Worms infected with*S. aureus* had a median survival of 7 days, and worms fed*E. coli* OP50 had a median survival of 8 days. Differences between curves were statistically significant (Log-rank test and Gehan–Breslow–Wilcoxon test; *p* < 0.0007). (D) *P. aeruginosa* infection of high-glucose-fed worms. Worms infected with*P. aeruginosa* had a median survival of 7 days, and worms fed *E. coli*OP50 had a median survival of 8 days. Differences between curves were statistically significant (Log-rank test and Gehan–Breslow–Wilcoxon test; *p* < 0.0007). (E) Worms were infected with*S. aureus* after being fed a high-glucose diet and after being fed the control diet. High-glucose worms had a median survival of 7 days, and control-fed worms had a median survival of 6 days. Differences between curves were statistically significant; Log-rank test (*p*: 0.006) and Gehan–Breslow–Wilcoxon test (*p*: 0.0027). (F) Worms were infected with*P. aeruginosa* after being fed a high-glucose diet and after being fed the control diet. High-glucose worms had a median survival of 7 days, and control-fed worms had a median survival of 6 days. Differences between curves were not statistically significant; Log-rank test (*p*: 0.0702) and Gehan–Breslow–Wilcoxon test (*p*: 0.0802). Four technical replicates were performed in three independent assays (*N* = ∼180).

The comparison between groups showed that worms previously fed the high-glucose diet before the infection assay were more resistant to infection by both pathogens than worms fed the control diet. However, only the*S. aureus* infection comparison was statistically significant following the Holm-Bonferroni corrections (Figures 10E and 8F). The Kaplan–Meier curve analysis showed that worms infected with *S. aureus* or*P. aeruginosa* had a median survival of 6 days when fed the control diet, while the worms fed the high-glucose diet had a median survival of 7 days. On the last day of the assay (day 9), ∼5% of the worms were still alive with both diets in the *S. aureus* infection, while in the *P. aeruginosa* infection, no worms survived with the control diet, and ∼2% were still alive with the glucose diet.

When we evaluated the intestinal barrier function of worms (Figure 11A) using erioglaucine, on day three of*S. aureus* infection, similar to the usual feeding with *E. coli* OP50 (Figure 11B), worms previously fed the high-glucose diet had a similar intestinal leakage profile to worms fed the control diet (Figure 11C). However, on day three of*P. aeruginosa* infection, worms previously fed the high-glucose diet had less intestinal leakage than worms previously fed the control diet (Figure 11D).

**Figure 11:**
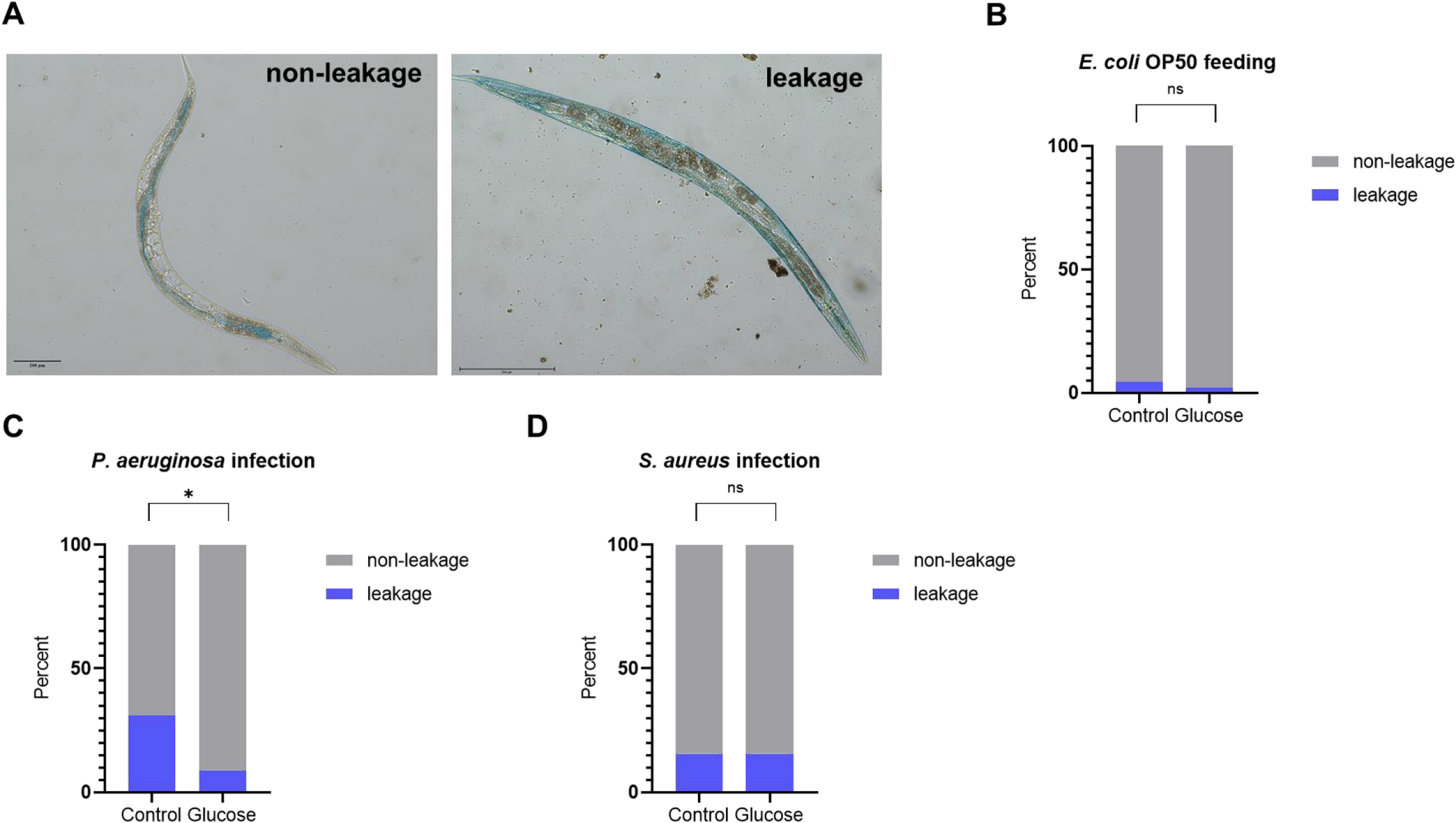
A high-glucose diet protects*C. elegans*’ intestinal barrier function from *P. aeruginosa* infection. Intestinal barrier function analysis of worms on day 3 of the infection assay, after being fed a high-glucose diet or a control diet. (A) Representative images of worms with nonleakage and leakage profiles; Scale bar: 200 μm. (B) Control worms, feeding on*E. coli*OP50, (C) worms infected with *S. aureus*, and (D) worms infected with*P. aeruginosa*. Fifteen worms from each group, from three independent assays, were photographed (*N* = 45). A percentage of worms with leakage was calculated. Comparison between the high-glucose diet (Glucose) and the control diet (Control) groups was performed using Fisher’s exact test. ns: not significant; *p: 0.0161.

Additionally, we performed a survival assay using the*C. elegans* strain RB1573, which lacks the immunity-related gene *dod-22* (Downstream Of DAF-16; WBGene00010125) and is associated with defense against Gram-negative bacteria. When we compared RB1573-infected worms to the N2 strain, Kaplan–Meier curve analysis showed that N2 worms survived longer than RB1573 worms during*P. aeruginosa*infection, confirming the role of this gene in worm defense to a Gram-negative pathogen (Figure 12A). RB1573 worms previously fed the high-glucose diet survived longer than those fed the control diet (Figure 12B). Moreover, N2 worms previously fed the high-glucose diet also survived longer than RB1573 worms fed the same diet (Figure 12C). In the worms infected by*S. aureus*, RB1573 worms survived more than N2 worms (Figure 12D) and no statistically differences in the survival of RB1573 worms previously fed a high-glucose diet and worms fed the control diet was observed (Figure 12E).

**Figure 12:**
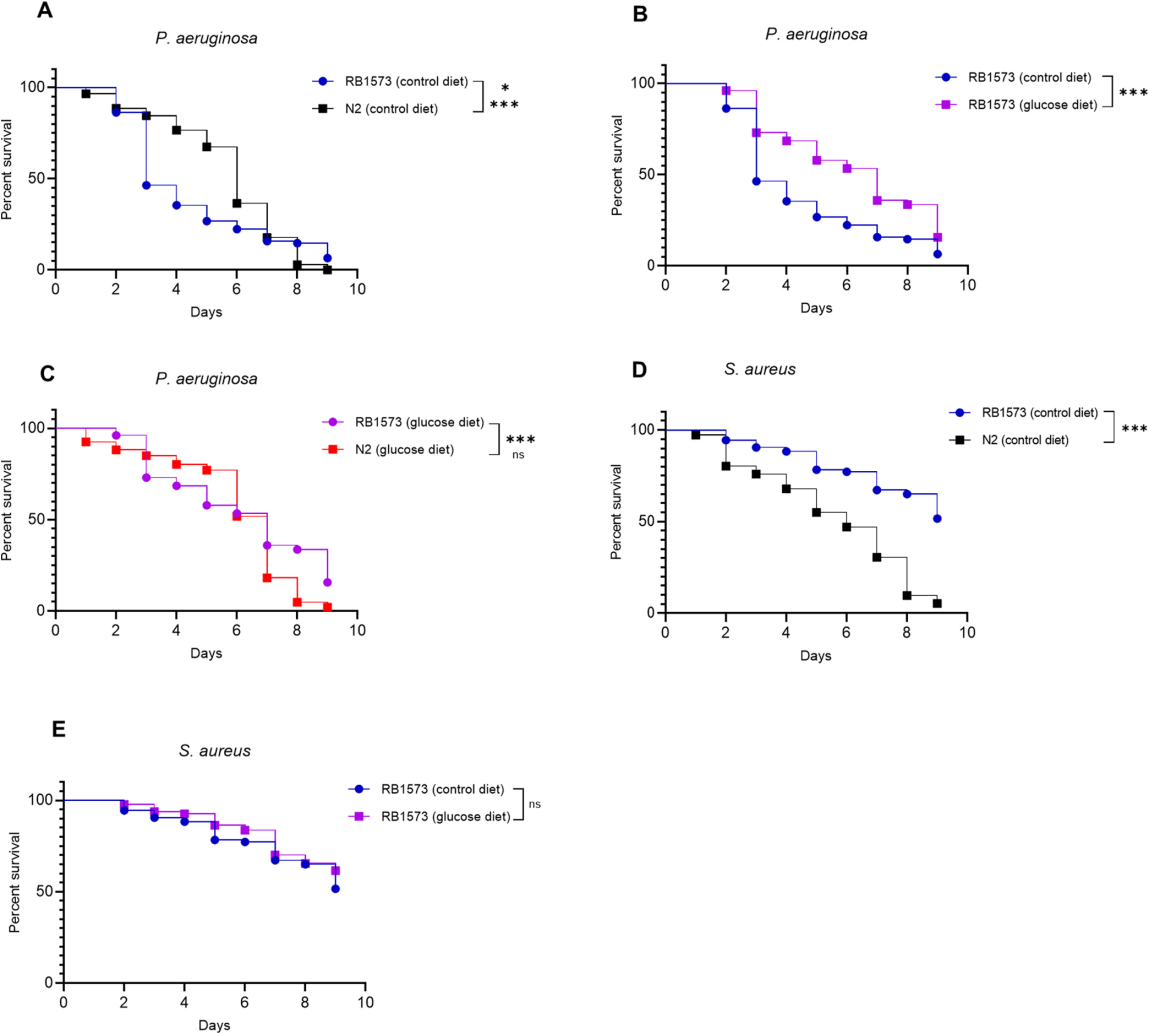
A high-glucose diet enhances the survival of the immune-deficient*C. elegans* RB1573 strain infected with*P. aeruginosa*. (A) N2 and RB1573 (*dod-22* deletion) worms were fed the control diet and infected with *P. aeruginosa* (B) RB1573 worms previously fed a high-glucose diet compared to RB1573 worms fed the control diet after *P. aeruginosa* infection (C) N2 and RB1573 were previously fed the high-glucose diet and infected with *P. aeruginosa* (D) N2 and RB1573 worms infected with *S. aureus* (control diet). (E) RB1573 worms previously fed a high-glucose diet compared to RB1573 worms fed the control diet after *S. aureus* infection. Four technical replicates were performed in three independent assays (*N* = ∼180). Survival curves were compared using Log-rank (Mantel-Cox) and Gehan–Breslow–Wilcoxon tests. *P*-values were adjusted for multiple comparisons using the Holm-Bonferroni method; ns: not significant; *p: 0.0312; ***p: < 0.0004.

## 3 Discussion

Although the effects of a high-glucose diet on*C. elegans* have been studied, the previous work utilized various glucose concentrations (0.5 to 8%) and different stages of worm development,^3,18–24^ making direct comparisons challenging. Therefore, in the present study, we used a glucose concentration of 2% (110 mM), one of the most commonly applied concentrations in*C. elegans* research,^3,29^ and exposed worms to this diet from the L1 to the L4 developmental stages. After the high-glucose diet feeding, worms were exposed to*S. aureus* and*P. aeruginosa*, and for both bacterial infections, glucose protective effects were observed.

In the*C. elegans*N2*S. aureus* infection assay, worms previously exposed to a high-glucose diet exhibited significantly increased survival compared to control-fed counterparts. While the difference in the*P. aeruginosa* infection assay did not reach the adjusted threshold for statistical significance, a consistent trend toward a protective effect was observed, with high-glucose survival curves remaining qualitatively superior to control diets. Critically, the observed one-day increase in median survival represents a substantial biological shift given the nematode’s brief lifespan. Furthermore, the Mantel–Haenszel confidence interval (1.021–1.761) did not encompass unity, indicating a potential reduction in relative risk and supporting the presence of a biologically relevant effect. This hypothesis is further reinforced by our findings regarding prior glucose feeding preserved intestinal integrity during*P. aeruginosa*infection and significantly improved survival in the RB1573 mutant strain, suggesting that early glucose exposure enhances resistance to this bacterium.

Furthermore, we also evaluated how the high-glucose diet affects key biological functions of*C. elegans*, such as pharyngeal pumping, defecation, body size, reproduction, and the neuronal dopaminergic system. Measuring pumping per minute makes it possible to identify food intake differences between diets or food intake problems.^30^ We did not find any statistical differences in pharyngeal pumping between worms fed the high-glucose and the control diet. This suggests that the other effects observed with high-glucose feeding were not solely due to problems with food intake, especially the bacterial infections that rely on the worms’ feeding on pathogens.

However, upon evaluating the defecation cycle, it was observed that worms subjected to a high-glucose diet exhibited a reduction in the duration of the defecation cycle. Alterations in the defecation cycle can influence the infection pattern in worms.^31^ Although it cannot be confirmed whether the effect of glucose persists after the worms are transferred from glucose plates to the survival assay, an increase in defecation may account for the differences observed in survival rates between control worms and those fed with glucose, particularly during the initial days of infection. An increased frequency of defecation contractions could potentially impair bacterial colonization within the intestinal tract.

Another key biological function affected by high glucose intake was body size. Previous work also found differences in body size when*C. elegans*were fed glucose. De Guzman et al.^32^ fed worms with 250 mM of glucose and found a significant 10% increase in body length (compared to worms without a glucose diet) in day-one adult worms. Worms (L1 to L4) fed glucose (100 mM) were longer and thicker than worms not fed glucose in the work of Alcántar-Fernández et al.^3^

The high-glucose diet also influenced brood size. Worms fed the high-glucose diet produced less progeny than the control worms. These results are in agreement with the work of Salim and Rajini,^33^ in which they used the same glucose concentration in worms from the egg stage to L4, and found a 36% reduction in egg-laying compared to the control. Reduced reproduction was also found in the work of Morton et al.^29^ in which adults worms fed high-glucose (100 mM) had a 17% mean reduction in egg-laying compared to control worms. However, Alcántar-Fernández et al.^3^ using L1 to L4 worms, and Teshiba et al.^34^ using L4 worms did not observe a reduction in egg-laying of worms fed glucose (100 mM).

The nervous system of*C. elegans* controls various biological functions within the worm’s organism, including immune responses. Multiple neurotransmitter pathways, such as acetylcholine, octopamine, and dopamine, are involved in the intestinal defense against bacterial pathogens.^35,36^ We observed that a high-glucose diet increased the prevalence of animals exhibiting neuronal damage. De Guzman et al.^32^ also found evidence of neuron degeneration in adult worms fed a high-glucose diet through analysis of fluorescence intensity loss. However, they used a higher glucose concentration (250 mM). Late L4 worms fed 400 mM of glucose had a 40% reduction in fluorescent signaling in the work of Pinkas et al.^22^ Interestingly, Morton et al.^29^ showed that the glucose diet (100 mM) in adult worms did not induce neurodegeneration in *C. elegans*.

Besides, according to the study of Cao and Aballay,^13^ dopamine signaling can control immunity by regulating the p38 MAP kinase activity. The study showed that dopaminergic neurons regulate the*C. elegans* intestinal response to*P. aeruginosa* and that their ablation rendered worms more resistant to*P. aeruginosa*. So, in our work, over half of the worms that were fed glucose showed damage to their dopaminergic neurons. Then, after being exposed to*P. aeruginosa* and*S. aureus*, it could be hypothesized that dopamine signaling could help the worms become more tolerant to these pathogens compared to the control worms that had less dopaminergic neuron damage.

Considering the organismal changes induced by a high-glucose diet that might influence worms’ tolerance to*P. aeruginosa* and *S. aureus* infections, we further investigate lipid droplet abundance and oxidative stress responses generated by the high-glucose diet. Since*C. elegans* does not have adipose tissue, excess energy caused by a high-glucose diet can be stored as lipid droplets in intestinal and subcutaneous tissue cells.^37^ During pathogen infection, the worm’s entire metabolism is altered to respond to the damage caused by the pathogens, including lipid resource allocation.^38^ We found that worms fed a high-glucose diet had more lipid droplet abundance than the control worms. Therefore, when worms were infected by*S. aureus* and*P. aeruginosa*, those that had previously been fed the high-glucose diet had more lipid resources to use and reallocate to respond to pathogen damage and consequently survived more than the control worms.

In pathogenic infections,*C. elegans* produces ROS as part of its innate defense response. However, pathogen-induced cellular damage also contributes to ROS generation.^28^ Therefore, the induction of antioxidant enzymes is essential for maintaining redox homeostasis and protecting the worms from infection. In the present study, we observed higher *gst-4* expression in worms fed a high-glucose diet compared with control worms. We speculate that the increased *gst-4* expression observed here may help mitigate ROS generation associated with bacterial infection.

When ROS production was evaluated over time, no statistically significant differences were observed between high-glucose-fed worms and control worms at the early time points (0 and 6 h). However, at 24 and 48 h, worms fed the high-glucose diet exhibited significantly lower ROS levels than controls. These findings differ from most studies investigating high-glucose diets, which typically report increased ROS production.^39–41^ Nevertheless, Wang et al.^42^ demonstrated that glucose can suppress ROS production and SKN-1 activity, whereas Morton et al.^29^ reported a protective effect of glucose in adult worms. The transcription factor SKN-1 (worm homologue of the mammalian transcription factor Nrf2) plays a central role in stress responses in*C. elegans*, including pathogen resistance, and regulates *gst-4* expression.^11,43^

The insulin/IGF-1 signaling pathway is a major regulator of *C. elegans* physiology and acts as a key upstream regulator of the transcription factor DAF-16, whereby increased insulin signaling reduces DAF-16 nuclear translocation.^10^ In addition, oxidative stress can promote DAF-16 nuclear localization. In the present study, although a slight increase in nuclear localization was observed in high-glucose-fed worms, no statistically significant differences were detected between worms fed a high-glucose diet and control-diet-fed worms. These findings suggest that the effects of glucose exposure on oxidative stress responses in our experimental conditions may not be primarily mediated through major changes in DAF-16 activation, or that more subtle regulatory mechanisms may be involved.

Because worms were exposed to the high-glucose diet during early development and subsequently exhibited increased *gst-4* expression and enhanced resistance to bacterial infection, we can infer that glucose does not directly suppress SKN-1 activity in this context. Instead, early exposure to glucose may induce mild metabolic stress, enhancing the worms’ capacity to respond to subsequent challenges, potentially by facilitating SKN-1 activation. This adaptive response could promote increased *gst-4* expression, thereby reducing ROS levels and improving resistance to bacterial infection. Furthermore, although we used live*E. coli* OP50 as a nonpathogenic control, it can induce low levels of physiological damage in worms,.^44,45^ Thus, the early adaptive response to stress may explain the progressive increase in ROS observed in the control group and the divergence between the control and previously glucose-fed worms at later time points.

Other results supporting the hypothesis of an adaptive response to stress include findings on senescence. We monitored the formation of a blue pigment associated with AGE, a marker of senescence. Previous studies have shown that a high-glucose diet enhances AGE formation.^39–41^ In our study, we found a difference in blue pigment levels between high-glucose-fed and control-fed worms at 48 h. Control worms showed higher pigment formation than high-glucose worms, indicating that worms previously fed the glucose diet, even after 48 h, respond better to age stress.

Similar to this hypothesis, Tauffenberger et al.^46^ investigated the effects of a high-glucose diet on*C. elegans* lifespan and found that a high-glucose diet during early development extended lifespan, whereas a high-glucose diet during adulthood reduced lifespan. According to Moreno-Arriola et al.^47^ ROS has already been described as signaling molecules for adaptive responses in humans and*C. elegans*.

Taken together, further studies are required to evaluate SKN-1 expression and the immune effectors regulated by its activation in order to confirm this proposed mechanism. Additionally, this study has some limitations that should be acknowledged. We did not assess oxidative stress in earlier developmental stages, such as L1 larvae, or adult-feeding groups, which may provide important insights into the initial effects of glucose exposure and the onset of adaptive stress responses. Moreover, ROS production was not evaluated during bacterial infection, preventing a direct assessment of the dynamic interaction among glucose exposure, pathogen-induced oxidative stress, and host defense mechanisms. Addressing these aspects in future studies will be essential to better elucidate the role of early glucose exposure in redox regulation and immune responses in*C. elegans*.

The bacterial infection resistance observed after a high-glucose diet in our study was similar to that reported by Lavigne et al.^25^ They investigated the influence of the glucose diet on bacterial infection by performing a solid-killing assay of*C. elegans* DH26 (*fer-15* (b26)­II; fertile at 15 °C and sterile at 25 °C) infected with*E. coli* and*S. aureus* clinical isolates. The authors used L4 stage worms and performed infection on the glucose plates. Despite differences in assay conditions, these authors also found that worms fed a high-glucose (2%) diet were more resistant to infection by both pathogens. We used a liquid medium survival assay and did not observe growth of *S. aureus* in the presence of glucose, as the worms had been fed glucose prior to the assay. Therefore, the increased survival rate of worms fed the high-glucose diet can be associated more with the effect of glucose uptake than the effect of glucose on*S. aureus* growth.

Lavigne et al.^25^ performed infection assays in *C. elegans* *daf-16* mutants and observed no significant change in susceptibility to pathogens. The authors attributed this finding to the functional redundancy of DAF-16 in *C. elegans*, suggesting that additional immune regulators may compensate for its loss. Consistent with this hypothesis, we evaluated a strain lacking *dod-22*, a downstream target of DAF-16. In our experiments, worms deficient in *dod-22* showed no increased susceptibility to *S. aureus* infection and, in fact, exhibited improved survival compared with N2 worms. When RB1573 worms were previously fed the high-glucose diet, no statistically significant differences were found compared to control-fed worms. We infer that other immunity pathways that act in parallel to DAF-16 are more effective at protecting worms from *S. aureus* infection. The p38 MAP kinase pathway and the transcription factor HLH-30 have been described to act in worms’ defense against*S. aureus*, and could be great candidates to be overstimulated after glucose feeding.^48^

The *dod-22* gene has been previously implicated in the immune response of*C. elegans* to Gram-negative pathogens.^49^ This role was supported by our survival assays using the RB1573 mutant strain, which lacks *dod-22*, and comparing its response to *P. aeruginosa* infection with that of the N2 strain. Under a standard diet, the absence of *dod-22* reduced worm survival following *P. aeruginosa* infection, confirming its contribution to host defense against this pathogen. However, the protective effect of prior glucose exposure on survival was not abolished in the mutant strain, indicating that glucose-mediated protection against *P. aeruginosa* does not depend exclusively on this immune effector. These results further suggest that glucose exposure may enhance host resistance through alternative or complementary immune pathways, such as p38 MAP kinase pathway.^50^

Although the results were similar, our study differs from Lavigne et al. in the administration of the high-glucose diet: they used L4 worms for glucose and bacterial exposure, whereas we fed glucose to worms from the L1 to L4 stages and then performed an infection assay without glucose. That was also the difference between our work and that of Li et al. When Li et al.^26^ used 0.5% glucose to feed worms and performed a solid media killing assay with*S. typhimurium*, they observed opposite results in which the worms fed a glucose diet were more susceptible to *S. typhimurium* infection than the control worms. The authors attributed this to glucose-mediated inhibition of SKN-1. The difference in glucose administration could corroborate the early adaptation hypothesis discussed in our results regarding ROS production. Li et al. also performed their assays at a different glucose concentration (0.5%), and a limitation of our study is that we did not investigate other glucose concentrations, particularly with respect to the bacterial infection response.

Furthermore, when we evaluated intestinal barrier function in infected worms, we observed differences among the pathogens as early as day three. While no differences were found in*S. aureus* infection, in*P. aeruginosa* infection, worms previously fed the high-glucose diet had less intestinal leakage than worms previously fed the control diet. Despite our findings showing protective effects of the high-glucose diet with both*S. aureus* and*P. aeruginosa* infections, it appears that the effect of a high-glucose diet on*C. elegans* susceptibility to bacterial infection also depends on the type (or species) of bacteria infecting the worm. To date, our study is the first to investigate the effect of a high-glucose diet on*P. aeruginosa*infection in*C. elegans*.

Since we cannot separate*E. coli*from glucose during worm feeding, we cannot exclude an effect of this bacterium on the results observed. Considering*E. coli* as worms’ microbiota, Kingsley et al.^51^ aimed to isolate the effects of the microbiota using live bacteria precultivated with glucose. Their results showed reduced lifespan, higher ROS production, *gst-4* upregulation, changes in immunity, and higher susceptibility to infection with*P. aeruginosa*,*S. aureus*, and *Enterococcus faecalis*. However, we found it difficult to compare the bacterial assays, since we do not use glucose in our infection assay.

Overall, this work provides new data linking diet to infection resistance in*C. elegans*, showing that high-glucose feeding during early development alters resistance to bacterial infection, suggesting that the host metabolic state can modulate innate immune responses.*C. elegans*key metabolic and innate immune pathways are highly conserved across evolution, in which stress-response programs have strong functional parallels in mammals.^9^ Thus, this study provides a foundation for future work that allows information for mammalian systems, particularly in the context of metabolic disorders such as diabetes and infection susceptibility. Therefore, further research into the molecular mechanisms underlying the protective effects of a high-glucose diet against bacterial pathogens is essential to clarify the roles of cellular signaling pathways and metabolic stress.

In summary, our results indicated that alterations in the defecation cycle, dopaminergic neuron health, lipid metabolism, and oxidative stress responses can contribute to the enhanced survival of*C. elegans* after glucose feeding. Notably, to date, this is the first study to perform *S. aureus* and *P. aeruginosa* infections in the RB1573 strain, and the first to associate protective effects of a high-glucose diet with*P. aeruginosa*infection in*C. elegans*.

## 4 Methods

### 4.1 *C. elegans* Strains and Synchronization

*C. elegans* wild type N2 Bristol, CL2166 (*dvIs19* [(pAF15)*gst-4p*:GFP:NLS]), TJ356 (*zIs356* [*daf-16p*:*daf-16a/b*:GFP + rol-6­(su1006)]), BY200 (P_*dat‑1*_::GFP), and RB1573 (*dod-22* (*ok1918*) IV) were maintained on Nematode Growth Medium (NGM) seeded with *E. coli* OP50 at 20 °C.^52^ Synchronization to obtain worms at the same developmental stage was performed using an alkaline hypochlorite solution (“bleaching”).^53^

### 4.2 High-Glucose Diet

The high-glucose diet was prepared according to Alcántar-Fernández et al.,^3^ in which glucose was mixed with NGM to obtain a 2% concentration. The plates were seeded with live*E. coli* OP50. Nematodes maintained on NGM plates without glucose served as the control group (control diet). Approximately 1000–1500 worms from both control and glucose groups remained in plates from L1 until the L4 development stage. All assays described below were started with L4 worms.

### 4.3 Pharyngeal Pumping Rate Assay

Pharyngeal pumping was measured visually under EVOS FL Auto 2 Imaging System (Thermo Fisher Scientific) (10× magnification). The pumps per minute count was evaluated by observing the contraction and relaxation of the worm terminal bulb of the pharynx (“pump”) for 10 s three times. The mean of these values was multiplied by six to obtain an estimated number of pharyngeal pumping per minute. Observation and counting of pharyngeal pumping were performed by two evaluators. Ten L4 worms were used per group in seven independent assays (*N* = 70).^54^

### 4.4 Defecation Cycle Assay

The defecation cycle is a peristaltic contraction that begins at the back of the animal, spreading anteriorly and generating the expulsion of feces. The mean time in seconds for three intervals between two defecation cycles was measured. Observation and measurement of the time of the defecation cycle were performed by two evaluators. Ten animals per group were analyzed in seven independent assays (*N* = 70).^55^

### 4.5 Body Size Measurements

To measure the lengths and widths of the worms, they were washed for the plates at least 3 times, paralyzed with levamisole (1 mM), and photographed with EVOS FL Auto 2 Imaging System (Thermo Fisher Scientific) microscope in a 10× magnification. The images of 20 worms from each group, in three independent assays, were analyzed with ImageJ software (*N* = 60).^54^

### 4.6 Brood Size Assay

Three L4 worms per group were transferred to new NGM plates seeded with*E. coli* OP50 daily until they ceased egg-laying. The offspring were counted daily. Four independent assays were performed (*N* = 12).^56^

### 4.7 Dopaminergic Neuron Damage Analysis

*C. elegans* BY200 (P_*dat‑1*_::GFP)^57^ was used for dopaminergic neuron analysis. Dopaminergic neuron damage was identified according to Bijwadia et al.^58^ Worms were washed three times with M9 buffer from the plates with or without glucose, paralyzed with levamisole (1 mM), and put onto a microscope slide covered with a coverslip to be photographed with EVOS FL Auto 2 Imaging System (Thermo Fisher Scientific). Thirty images were taken at 40× magnification, and the percentage of worms with any dopaminergic neuron damage was calculated for each group. Three independent assays were performed (*N* = 90).

### 4.8 Lipid Droplet Abundance

Lipid droplet quantification using the Nile Red staining was performed as described in the Stuhr et al.^59^ protocol. Worms were washed for the plates at least 3 times to remove*E. coli*OP50. First, approximately 200 worms were fixed in 40% isopropanol, then stained with a Nile red solution, and finally incubated for 2 h in the dark. After two washes in M9 buffer, 10 μL of the stained pellet was placed on a microscope slide and covered with a coverslip. Ten (10)× magnification images were taken with EVOS FL Auto 2 Imaging System (Thermo Fisher Scientific) and processed with ImageJ software. For each group, 20 worms were measured individually across four independent assays (*N* = 80). The area-corrected worm fluorescent intensity (CTCF) was calculated and normalized by the control for the quantitative analysis.

CTCF:(wormintegrateddensity)−(backgroundintegrateddensity)/wormarea

### 4.9 *gst-4* Fluorescence Expression

*C. elegans* strain CL2166 (*dvIs19* [(pAF15)*gst-4*p:GFP:NLS]) was used to evaluate the oxidative stress by *gst-4* expression. Worms were washed and placed on a microscopic slide in the same way as in Section 4.7, and 20 images were taken at 10× magnification. Image analysis was performed with ImageJ software. For each group, 20 worms were measured individually in three independent assays (*N* = 60). The *gst-4* fluorescent intensity was quantified by CTCF in the same manner as described in Section 4.8.^59^

### 4.10 DAF-16 Cellular Localization

To analyze the cellular localization of the transcription factor, DAF-16, the strain TJ356 (zIs356 [*daf-16p*:*daf-16a*/b:GFP + rol-6­(su1006)]) was used. Worms were placed on a microscopic slide in the same manner as in Section 4.7, and the images were taken at 20× magnification. Twenty images from each group were analyzed from three independent assays (*N* = 60). The DAF-16 cellular localization classification into cytoplasmic, intermediate, and nuclear was performed according to Guédon et al.^60^

### 4.11 ROS Production and Senescence Pigment Monitoring

Worms were washed from the plates, and 100 worms per well were pipetted into a 96-well plate with dark sides to avoid interference with fluorescence readings. Live*E. coli*OP50 was added to each well plate. For ROS production, 50 mM 2′,7′-dichlorodihydrofluorescein diacetate (H_2_DCFDA) was added.^61^ Readings were performed on a Thermo Scientific Varioskan LUX spectrophotometer every 5 min for 48 h at an excitation/emission wavelength of 490–540 nm. The senescence pigment was measured without any other added substance. Readings were performed on a Thermo Scientific Varioskan LUX spectrophotometer every 5 min for 48 h at an excitation/emission wavelength of 360/435 nm.^62^

For both analyses, we selected four time points: 0 h and 6 h, in which worms are still in L4; easy to link to other assays performed in this development stage, and 24 h and 48 h, the latter time points to infer a persistent effect and association with the bacterial infection assay. For both analyses, at least four technical replicates from 7 independent assays were performed (*N* = ∼2800).

### 4.12 Bacterial Infection Assay

For the inoculum in the liquid-killing survival assay, *P. aeruginosa* UCBPP-PA14 and*S. aureus* ATCC 25904 were cultivated in TSB (Soy Tryptone Broth) with an OD at 620 nm of 0.9–1.0 and 1.0–1.4, respectively, and incubated at 37 °C for 18–24 h. These growth conditions stimulate the production of virulence factors. With the same purpose, the*P. aeruginosa* culture was incubated a second time at 25 °C for 24 h.^63^

Liquid survival assays were performed in 96-well plates. Approximately 10–25 L4 worms (N2 or RB1573) of each group (glucose and control) were added to the plate wells containing M9 buffer supplemented with cholesterol (5 mg/mL). One μL of *P. aeruginosa* or *S. aureus* cultures were added to the wells containing the worms. 5-Fluoro-2′-deoxyuridine (FUDR; 50 μg/mL) was used to avoid animal progeny. The plates were incubated at 25 °C, and the number of live worms was counted over a period of 9 days. The animals were considered dead due to the total absence of movement. Live*E. coli* OP50 was used as a noninfected control for both groups. GFP-tagged*E. coli*OP50 and mCherry-tagged *P. aeruginosa* PA14 were used to image worms in the liquid assay and confirm bacteria intake. Four technical replicates were performed in three independent assays for all groups (*N* = ∼180).

### 4.13 Intestinal Barrier Function Assay

To assess pathogen-induced intestinal damage, an intestinal barrier function assay was performed according to the protocol described by Madhu et al.^64^ This method utilizes a 5% (w/v) erioglaucine disodium salt solution (blue dye) with a 3 h incubation period. This specific concentration enhances microscopic visualization, while the 3-h duration ensures sufficient time for the dye to diffuse throughout the intestine or leak into the body cavity in cases of barrier compromise. Thus, N2 infected worms (3 days postinfection) were washed at least 3 times with M9 buffer and incubated for 3 h with a mixture of erioglaucine and*E. coli*OP50. After at least 3 washes with M9, the worms were paralyzed with levamisole (1 mM), mounted on a microscope slide with a coverslip, and photographed using the EVOS FL Auto 2 Imaging System (Thermo Fisher Scientific). The presence of the blue dye outside the intestinal lumen indicates reduced intestinal integrity. Worms with this phenotype were classified as “leakage”; worms with no blue dye outside the lumen were classified as “nonleakage”. Fifteen worms from each group were photographed, and the leakage percentage was calculated. Three independent assays were performed (*N* = 45).

### 4.14 Statistical Analysis

All statistical analyses and graphs were performed using the software GraphPad Prism 8.0.2. Statistical tests are identified in the figure legend of each graph. For all tests, *P* values of <0.05 were considered statistically significant.
